# Left atrial appendage occlusion combined with cryoballoon or radiofrequency ablation: One-year follow-up comparison

**DOI:** 10.3389/fcvm.2023.1153158

**Published:** 2023-04-26

**Authors:** Yibo Ma, Lanyan Guo, Miaoyang Hu, Qun Yan, Haitao Liu, Fu Yi

**Affiliations:** Department of Cardiology, Xijing Hospital, Air Force Medical University, Shaanxi, China

**Keywords:** atrial fibrillation, left atrial appendage occlusion, cryoballoon ablation, radiofrequency ablation, peri-device leak

## Abstract

**Background:**

A one-stop procedure involving catheter ablation and left atrial appendage occlusion (LAAO) is an option for high-risk atrial fibrillation patients. Few studies have reported the efficacy and safety of cryoballoon ablation (CBA) combined with LAAO, and no studies have compared the combination of LAAO with CBA or radiofrequency ablation (RFA).

**Methods:**

A total of 112 patients were enrolled in the present study; 45 patients received CBA combined with LAAO (group 1), and 67 patients received RFA combined with LAAO (group 2). Patient follow-up was performed for 1 year to detect peri-device leaks (PDLs) and safety outcomes (defined as a composite of peri-procedural and follow-up adverse events).

**Results:**

The number of PDLs at the median 59 days follow-up was comparable between the two groups (33.3% in group 1 vs. 37.3% in group 2, *p* = 0.693). Safety outcomes were also comparable between the two groups (6.7% in group 1 vs. 7.5% in group 2, *p* = 1.000). Multivariable regression showed that PDLs risk and safety outcomes were all similar between the two groups. Subgroup analysis of PDLs indicated no significant differences. Follow-up safety outcomes were related to anticoagulant medication, and patients without PDLs were more likely to discontinue antithrombotic therapy. The total procedure and ablation times were all significantly shorter for group 1.

**Conclusion:**

When compared with left atrial appendage occlusion combined with radiofrequency, left atrial appendage occlusion combined with cryoballoon ablation has the same risk of peri-device leaks and safety outcomes, but the procedure time was significantly reduced.

## 1. Introduction

As a serious clinical arrhythmia, atrial fibrillation (AF) can lead to high incidence rates of disability and fatality. An investigation of the global burden of cardiovascular disease in 2022 indicated that AF has become the sixth leading cause of death among cardiovascular diseases (1). AF can lead to a 5-fold increase in risk of ischemic stroke (2). Anticoagulant therapy is currently the first-line treatment for AF thromboprophylaxis, but it also increases the clinical risk of major bleeding (2, 3). Previous autopsy reports showed that approximately 90% of thrombi in AF patients originate from the left atrial appendage (LAA) (4). Left atrial appendage occlusion (LAAO) has emerged as a non-drug thrombosis prevention strategy that isolates the common anatomic source of thrombi. Regarding the composite end points of death and thromboembolism, LAAO is not inferior to anticoagulants; moreover, LAAO yields better results than anticoagulants for non-procedure-related major bleeding (5–7). LAAO also has unique thromboprophylaxis advantages in high-risk AF patients.

A simultaneous procedure combining catheter ablation with LAAO, also called a one-stop procedure, has become an option for high-risk AF patients. This procedure allows for simultaneous rhythm control and thrombosis prevention without increasing the risk of peri-procedural severe adverse events (SAEs) (8). In addition, compared to two individual procedures at different stages, this one-stop procedure advantageously reduces the financial burden to patients (9). Radiofrequency ablation (RFA) and cryoballoon ablation (CBA) are the two most commonly used catheter ablation methods in clinical practice. The performance of RFA and CBA in paroxysmal AF and persistent AF has been confirmed by a large number of trials: CBA is not inferior to RFA in terms of AF recurrence and AF burden; the risk of SAEs is similar between the two methods; and procedure and ablation times are significantly shorter for CBA than RFA (10–12). The majority of studies have evaluated the safety and efficacy of RFA combined with LAAO, but only a few studies have focused on the clinical efficacy of CBA combined with LAAO (13–19). In addition, no controlled studies of LAAO combined with RFA or CBA have been published. Due to different injury mechanisms, coumadin ridge edema caused by RFA and CBA is also different; although the magnitude of damage is similar, the duration of CBA-associated coumadin ridge edema was shown to be longer (20). With this in mind, CBA combined with LAAO may increase the incidence of peri-device leak (PDL) compared with RFA combined with LAAO. Incomplete occlusion increases the risk of clinical ischemic stroke (21). Therefore, it is necessary to evaluate the relative advantages and disadvantages of CBA compared with RFA in LAAO population.

## 2. Materials and methods

### 2.1. Study populations

**Figure 1:**
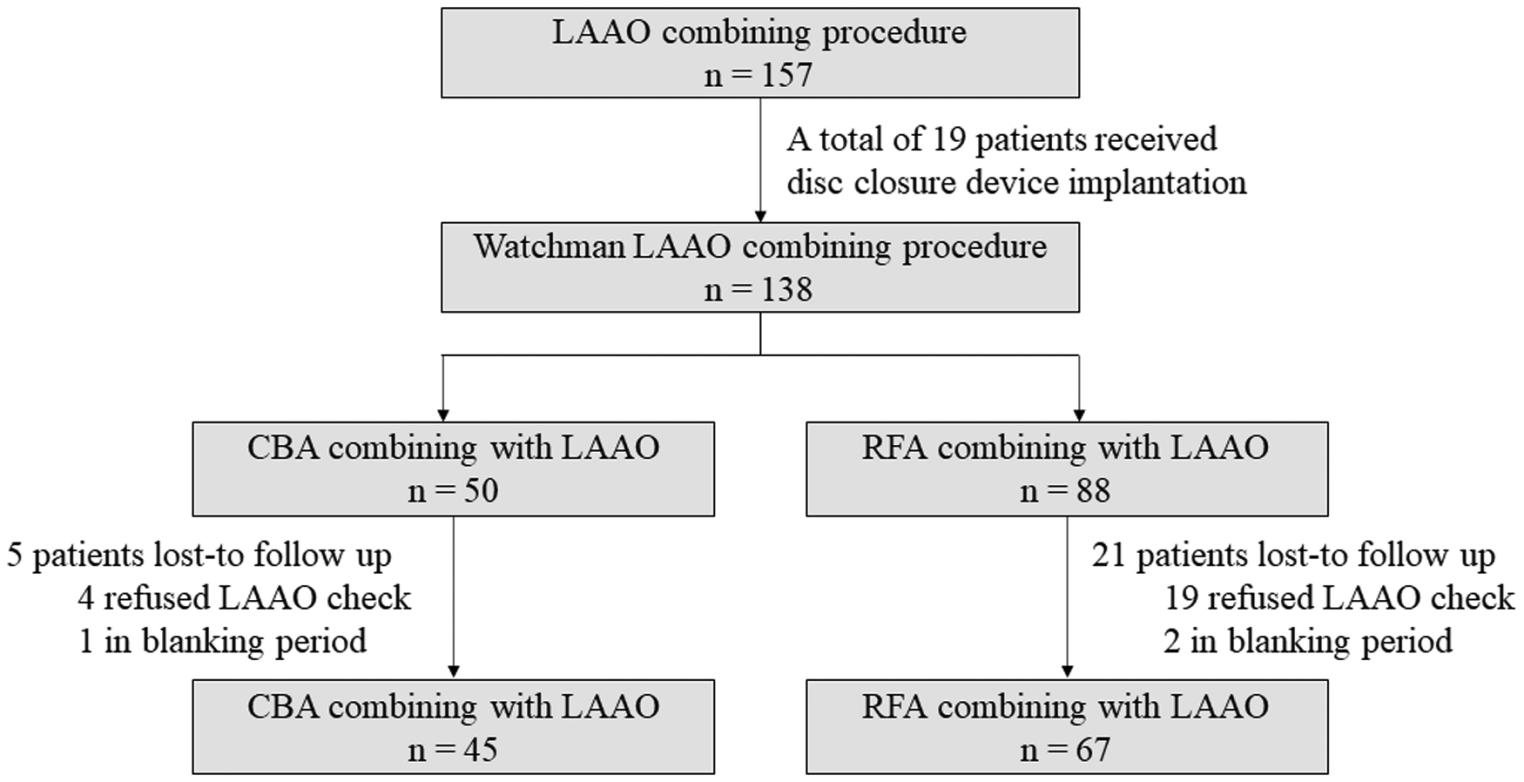
Study flowchart. LAAO, left atrial appendage occlusion; CBA, cryoballoon ablation; RFA, radiofrequency ablation.

This retrospective single-center study aims to prove the efficacy and safety of LAAO combined with CBA by comparing it to LAAO combined with RFA. A total of 157 patients who received LAAO at the First Affiliated Hospital of Air Force Medical University, Cardiology Department, between December 2019 and December 2021 were screened. For this study, the inclusion criteria were as follows: (1) age 18–85 years; (2) received CBA or RFA at the same time as LAAO; and (3) Watchman device (Boston Scientific, USA) implantation. The exclusion criteria were as follows: (1) valvular AF, defined as AF due to moderate or severe valve stenosis, surgical valve replacement, or surgical valve repairment; (2) cardiac surgery history; (3) received CBA and RFA simultaneously; (4) lost to follow-up in the first 3 months; or (5) any disc device implantation. A total of 112 patients were enrolled in this study. The study was approved by the Ethics Board of the First Affiliated Hospital of Air Force Medical University. Each patient provided their informed consent prior to the procedure. The details of the patient enrollment process are shown in Figure 1.

### 2.2. Procedure

#### 2.2.1. Cryoballoon ablation

CBA was performed as previously described (22). After establishing femoral access and puncturing the atrial septum, 100 U/kg heparin was administered to maintain an active clotting time of 250–350 s. Next, the cryoballoon was advanced into the left atrium. Protective pacing was performed first to evaluate the anatomical relationship between the phrenic nerve and left atrium. After that, the operator positioned the cryoballoon at the antrum of each pulmonary vein and filled the cryoballoon. Pulmonary vein occlusion was confirmed by venogram. If appropriate, ablation was performed for approximately 180 s, and the temperature was maintained at 45–55°C. If AF persisted after all ablations, cardioversion was performed to restore sinus rhythm. After 20 min of observation, we verified that pulmonary vein potentials were eliminated.

#### 2.2.2. Radiofrequency ablation

We performed ablation index-guided high power-short duration ablation (23). First, left atrial voltage mapping was performed. According to the voltage mapping, the operator performed pulmonary vein antrum isolation and left atrial substrate modification to isolate or eliminate low voltage areas. When ablating the anterior wall, the ablation index was set at 500, and the power was 45 W; when ablating the posterior wall, the ablation index was set at 400, and the power was 35 W. For the ablation of other sites, the ablation index was set at 450, and the power was 40 W. If AF persisted after all ablations, we performed cardioversion to restore sinus rhythm. After 20 min of observation, we verified bidirectional conduction blockage at each ablation lesion.

#### 2.2.3. Left atrial appendage occlusion

The ablation catheter was replaced with the device delivery sheath after ablation verification. A pigtail catheter was sent into the LAA through the device delivery sheath. Contrast medium was injected through the pigtail catheter to determine the morphology, orifice diameter, and depth of the LAA. Next, the pigtail catheter was removed, and the Watchman device catheter was advanced into the LAA. The device was deployed, and the occlusion was verified according to the PASS principle (Supplementary Table S1) (24). If appropriate, the operator released the device.

### 2.3. Follow-up

Out-patient follow-up was scheduled at 1, 2, 3, 6, 9, and 12 months post-procedure. During each follow-up visit, the patients were asked to receive an electrocardiogram and Holter monitoring to detect abnormal heart rhythms. To facilitate timely feedback, online follow-up was also available if patients experienced paresthesia, palpitations, or other discomfort. At months 2 and 12, the patients were required to undergo LAAO follow-up. Antithrombotic medication post-procedure was administered primarily according to the following recommended guidelines and instructions: for the first 2 months, oral anticoagulants with or without single antiplatelet therapy; for the next 4 months, dual antiplatelet therapy; and finally, lifelong single antiplatelet therapy (24, 25). Antiarrhythmic drugs were required to be taken at least 3 months post-procedure. Physicians tailored the medications to the patients' specific conditions.

### 2.4. End points

The primary efficacy outcome was PDL. PDL were defined as blood flow entering the distal end of the LAA along the closure device margin. According to the leakage size, we classified PDLs using 3 grades: <3 mm, 3–5 mm, and >5 mm (defined as occlusion failure). Both transesophageal echocardiography and computed tomography were available for leakage assessment. Arrhythmia recurrence as a secondary efficacy outcome was defined as any episode of atrial arrhythmia lasting more than 30 s occurring after the blanking period, with or without antiarrhythmic medications. The blanking period refers to the first 3 months post-procedure. Electrocardiogram, Holter device, emergency monitor, and continuous recorder were available for heart rhythm evaluation.

The safety outcome was a composite of adverse events during procedure and follow-up, including all-cause or cardiovascular death, stroke (ischemic or hemorrhage) or systemic embolism, major bleeding, cardiac tamponade, phrenic nerve paralysis, device or air embolization, and complete atrioventricular block. Puncture site complications or pericardial effusion served as additional peri-procedure safety events. Major bleeding was defined as Bleeding Academic Research Consortium (BARC) 3 or 5 bleeding (26).

### 2.5. Statistical analysis

We performed Shapiro-Wilk tests to explore whether continuous variables had normal or skewed distributions. For normal distributions, we used the mean ± SD for descriptions and one-way ANOVA for comparisons; for skewed distributions, we used the median (IQR) [range] for descriptions and Mann-Whitney U tests for comparisons. Categorical variables are described as counts (percentages) and compared with Fisher's exact test. Times to arrhythmia recurrence is described with Kaplan-Meier curve and compared with log-rank test. Changes in post-procedure medications for different follow-up periods are described with Sankey diagrams.

Multivariable logistic regression was used to explore whether PDL and safety outcomes were significantly different between the two combination procedures. Multivariable model 1 was adjusted for CHA_2_DS_2_-VASc and HASBLED scores. Multivariable model 2 was adjusted for demographic characteristics (age, sex, body mass index), type of AF, AF duration, commodities (heart failure, hypertension, diabetes, stroke, vascular disease), echocardiographic index (left atrial diameter, ejection fraction), and peri-procedure characteristics (left atrial appendage orifice, redo procedure). Multivariable model 3 was adjusted for covariables with group-to-group differences. We also used propensity score matching for a secondary analysis. This method was based on a logistic regression model. Patients were selected by using the 1:1 nearest neighbor matching method after adjusting covariables with group-to-group differences.

We performed subgroup analysis to determine group-to-group differences in PDLs among specific populations. Subgroup selection was determined by risk score suggested stratification, clinical experience, and potential risk factors suggested by univariable logistic regression. For ranked variables, we defined the median as their cut-off points; for continuous variables, we performed the restricted cubic spline to find the appropriate thresholds for both groups.

R 4.2.0 and IBM SPSS Statistics 26.0 were used to perform statistical analyses. Statistical significance was defined as two-sided *p* ≤ 0.05.

## 3. Results

### 3.1. Patients characteristics at baseline

A total of 112 patients were enrolled in the present study; 45 patients were in the LAAO combined with CBA group (group 1), and 67 patients were in the LAAO combined with RFA group (group 2). Baseline characteristics are listed in Table 1. Most characteristics were comparable between the two groups, except for higher HASBLED scores (1 (0, 2) [0, 4] vs. 2 (1, 2) [0, 4], *p* = 0.036) and larger left atrial diameters (41.2 ± 5.5 vs. 44.9 ± 5.4, *p* = 0.001) in group 2. Over half of the patients presented with non-paroxysmal AF. The median CHA_2_DS_2_-VASc was 3 (2, 4) [0, 7]. A total of 18 (16.1%) patients had a history of cardiac embolism (15.6% in group 1 vs. 16.4% in group 2, *p* = 1.000).

**Table 1:** Patient characteristics at baseline.

	Group 1 (*n* = 45)	Group 2 (*n* = 67)	*P* value
**Demographic characteristics**			
Age, years	61.4 ± 9.8	63.7 ± 8.4	0.199
Height, m	167.0 ± 8.1	168.3 ± 7.5	0.384
Weight, kg	68.7 ± 10.4	71.9 ± 11.7	0.143
Body mass index, kg/m^2^	24.6 ± 3.0	25.3 ± 3.2	0.227
**AF overview**			
Type			0.324
Paroxysmal, *n* (%)	20 (44.4)	23 (34.3)	–
Non-paroxysmal, *n* (%)	25 (55.6)	44 (65.7)	–
Time to first diagnosis, months	24 (4, 48) [1, 240]	12 (3, 48) [1, 240]	0.594
**Commodities or risk factors**			
CHA_2_DS_2_-VASc score	3, (1, 4) [0, 7]	3 (2, 4) [1, 7]	0.134
HASBLED score	1 (0, 2) [0, 4]	2 (1, 2) [0, 4]	0.036
Heart failure, *n* (%)	10 (22.2)	21 (31.3)	0.389
Hypertension, *n* (%)	17 (37.8)	37 (55.2)	0.084
Age ≥ 75 years, *n* (%)	3 (6.7)	4 (6.0)	1.000
Diabetes, *n* (%)	4 (8.9)	11 (16.4)	0.397
Systemic embolism, *n* (%)	9 (20.0)	17 (25.4)	0.649
Vascular disease, *n* (%)	39 (86.7)	49 (73.1)	0.104
Coronary artery disease, *n* (%)	6 (13.3)	19 (28.4)	0.068
Age 65–74 years, *n* (%)	14 (31.1)	32 (47.8)	0.117
Female, *n* (%)	19 (47.5)	21 (31.3)	0.315
**Examinations**			
Left atrial diameter, mm	41.2 ± 5.5	44.9 ± 5.4	0.001
Ejection fraction, %	57.0 (55.5, 60.0)	57.0 (54.0, 59.0)	0.354
Pathological regurgitation			
Mitral regurgitation, *n* (%)	4 (8.9)	11 (16.4)	0.397
Tricuspid regurgitation, *n* (%)	11 (24.4)	20 (29.9)	0.667
HbO_2_, g/l	144.1 ± 13.6	149.0 ± 15.0	0.078
Alanine transaminase, U/L	21.0 (16.5, 33.0)	22.0 (14.0, 29.0)	0.603
Aspartate aminotransferase, U/L	23.0 (17.5, 30.0)	21.0 (18.0, 24.0)	0.275
NT-proBNP, pg/ml	681.6 (250.0, 1,172.0)	558.5 (228.5, 994.6)	0.587
Serum Ca^+^, mmol/L	2.3 ± 0.1	2.3 ± 0.1	0.762
Glucose, mmol/L	5.5 (5.0, 6.2)	5.6 (5.1, 6.7)	0.267
LDL-C, mmol/L	1.9 (1.5, 2.5)	1.7 (1.4, 2.7)	0.353
HDL-C, mmol/L	1.2 (1.0, 1.3)	1.1 (0.9, 1.3)	0.068
TG, mmol/L	1.2 (0.9, 1.7)	1.2 (0.9, 1.7)	0.976

Data were described as mean ± SD, median (IQR) or *n* (%).

AF, atrial fibrillation; LDL-C, low density lipoprotein cholesterol; HDL-C, high density lipoprotein cholesterol; TG, total triglycerides.

### 3.2. Peri-procedure characteristics

Peri-procedure characteristics are listed in Table 2. The morphology, orifice diameter, and LAA depth were comparable between the two groups. The most common LAA morphology was cauliflower, and the most commonly used device was 27 mm in size. Three (4.5%) patients in group 2 received a redo procedure. The incidence rates of device reselection (4.4% in group 1 vs. 1.5% in group 2, *p* = 0.563) and redeployment (26.7% in group 1 vs. 37.3% in group 2, *p* = 0.307) were comparable between the two groups. All patients had successful device implantation, and 8 patients in each group had < 5 mm PDLs (17.8% vs. 11.9%, *p* = 0.419). The total procedure time was significantly shorter for group 1 (130.0 (120.0, 155.0) min vs. 245.0 (217.5, 300.0) min, p = 0.000), as well as the times for catheter ablation (112.0 (104.0, 138.0) min vs. 224.0 (204.5, 270.0) min, *p* = 0.000). The ablation details are shown in Supplementary Table S2. Length of stay post-procedure was similar between the two groups, and all patients received antithrombotic therapy.

**Table 2:** Periprocedural characteristics.

	Group 1 (*n* = 45)	Group 2 (*n* = 67)	*P* value
Left atrial appendage morphology			0.972
Cauliflower, *n* (%)	35 (77.8)	48 (71.6)	–
Chicken wing, *n* (%)	4 (8.9)	7 (10.4)	–
Reversed chicken wing, *n* (%)	1 (2.2)	2 (3.0)	–
Windsock, *n* (%)	2 (4.4)	3 (4.5)	–
Cactus, *n* (%)	3 (6.7)	7 (10.4)	–
Appendage ostia diameter, mm	21.0 ± 3.8	21.7 ± 3.2	0.283
Appendage depth, mm	22.4 ± 4.1	22.6 ± 3.7	0.806
Redo ablation, *n* (%)	0 (0.0)	3 (4.5)	0.272
Device size, mm	27 (24, 30)	27 (24, 30)	0.658
21 mm	10 (22.2)	6 (9.0)	0.058
24 mm	8 (17.8)	18 (26.9)	0.362
27 mm	13 (28.9)	25 (37.3)	0.418
30 mm	11 (24.4)	14 (20.9)	0.652
33 mm	3 (6.7)	4 (6.0)	1.000
Device reselection, *n* (%)	2 (4.4)	1 (1.5)	0.563
Redeployment, *n* (%)	12 (26.7)	25 (37.3)	0.307
1, *n* (%)	9 (20.0)	17 (25.4)	0.649
≥2, *n* (%)	3 (6.7)	8 (11.9)	0.521
Peri-device leak, *n* (%)	8 (17.8)	8 (11.9)	0.419
Jet <3 mm, *n* (%)	6 (13.3)	5 (7.5)	0.344
Jet 3–5 mm, *n* (%)	2 (4.4)	3 (4.5)	1.000
Total time spent, min	130.0 (120.0, 155.0)	245.0 (217.5, 300.0)	0.000
Ablation time spent, min	112.0 (104.0, 138.0)	224.0 (204.5, 270.0)	0.000
In hospital post-procedure, days	2 (2, 3)	2 (2, 3)	0.586

Data were described as mean ± SD, median (IQR) or *n* (%).

### 3.3. Follow-up peri-device leaks

After the median 59 (52, 71) days of follow-up, all patients received an LAAO examination. The details are listed in Table 3. The PDL incidence was comparable between the two groups (33.3% in group 1 vs. 37.3% in group 2, *p* = 0.693). Successful implantation was achieved similarly between the two groups (100.0% vs. 97.0%, *p* = 0.515). The incidence of <3 mm leakage was comparable between the two groups (26.7% vs. 17.9%, *p* = 0.348), while 3–5 mm leakage was numerically smaller for group 1 (6.7% vs. 16.4%, *p* = 0.154). In addition, both the newly-developed PDL (15.6% in group 1 vs. 25.4% in group 2, *p* = 0.248) and the progressed PDL (17.8% in group 1 vs. 29.9% in group 2, *p* = 0.184) were all comparable between the two groups. However, in group 1, both the >3 mm newly-developed PDL (0.0% vs. 10.4%, *p* = 0.040) and >3 mm progressed PDL (2.2% vs. 14.9%, *p* = 0.048) were significantly lower than in group 2. During follow-up, one (2.2%) patient (with a PDL of 2.5 mm) in group 1 experienced device-related thrombosis due to premature anticoagulant discontinuance.

**Table 3:** Follow-up LAAO results.

	Group 1 (*n* = 45)	Group 2 (*n* = 67)	*P* value
Examinations			0.248
TEE, *n* (%)	27 (60.0)	32 (47.8)	–
CCTA, *n* (%)	18 (40.0)	35 (52.2)	–
LAAO success, *n* (%)	45 (100.0)	65 (97.0)	0.515
Peri-device leaks, *n* (%)	15 (33.3)	25 (37.3)	0.693
Jet <3 mm, *n* (%)	12 (26.7)	12 (17.9)	0.348
Jet 3–5 mm, *n* (%)	3 (6.7)	11 (16.4)	0.154
Newly-developed peri-device leaks, *n* (%)	7 (15.6)	17 (25.4)	0.248
Jet <3 mm, *n* (%)	7 (15.6)	10 (14.9)	1.000
Jet 3–5 mm, *n* (%)	0 (0.0)	5 (7.5)	0.081
Jet >5 mm, *n* (%)	0 (0.0)	2 (3.0)	0.515
Persistent peri-device leaks, *n* (%)	8 (17.8)	8 (11.9)	0.419
Jet <3 mm, *n* (%)	5 (11.1)	2 (3.0)	0.115
Jet 3–5 mm, *n* (%)	3 (6.7)	6 (9.0)	0.738
Progressed peri-device leaks, *n* (%)	8 (17.8)	20 (29.9)	0.184
Jet <3 mm, *n* (%)	7 (15.6)	10 (14.9)	1.000
Jet 3–5 mm, *n* (%)	1 (2.2)	8 (11.9)	0.082
Jet > 5 mm, *n* (%)	0 (0.0)	2 (3.0)	0.515
Device-related thrombosis, *n* (%)	1 (2.2)	0 (0.0)	0.402

Data were described as *n* (%).

TEE, transesophageal echocardiography; CCTA, cardiac computed tomography angiography; LAAO, left atrial appendage occlusion.

### 3.4. Peri-procedure and follow-up safety

A total of 8 (7.1%) patients experienced safety outcomes; 3 (6.7%) patients were from group 1, and 5 (7.5%) patients were from group 2 (*p* = 1.000). All 3 patients from group 1 experienced procedure-related complications (2 had hematomas, and 1 had mild pericardial effusion); from group 2, 4 (6.0%) patients experienced procedure-related complications (2 experienced major bleeding requiring transfusion, 1 had a hematoma with BARC 3b major bleeding, and 1 had a pseudoaneurysm), and 1 (1.5%) patient experienced BARC 3a major bleeding (oral hemorrhage) due to anticoagulant treatment 3 months post-procedure. No patients died, and none of the patients experienced stroke, cardiac tamponade, or phrenic nerve paralysis peri-procedure or during follow-up (Table 4).

**Table 4:** Peri-procedural and follow-up safety.

	Group 1 (*n* = 45)	Group 2 (*n* = 67)	*P* value
**Peri-procedural safety**			
Total, *n* (%)	3 (6.7)	4 (6.0)	1.000
Major bleeding, *n* (%)	0 (0.0)	3 (4.5)	0.272
Systemic embolism, *n* (%)	0 (0.0)	0 (0.0)	1.000
Cardiac tamponade, *n* (%)	0 (0.0)	0 (0.0)	1.000
Hematoma, *n* (%)	2 (4.4)	1 (1.5)^†^	0.563
Pseudoaneurysm, *n* (%)	0 (0.0)	1 (1.5)	1.000
Pericardial effusion, *n* (%)	1 (2.2)	0 (0.0)	0.402
**Follow-up safety**			
Total, *n* (%)	0 (0.0)	1 (1.5)	1.000
Death, *n* (%)	0 (0.0)	0 (0.0)	1.000
Major bleeding, *n* (%)	0 (0.0)	1 (1.5)	1.000
Systemic embolism, *n* (%)	0 (0.0)	0 (0.0)	1.000
Delayed cardiac tamponade, *n* (%)	0 (0.0)	0 (0.0)	1.000

Data were described as *n* (%).

^†^ This patient experienced major bleeding due to hematoma.

### 3.5. Arrhythmia recurrence

**Figure 2:**
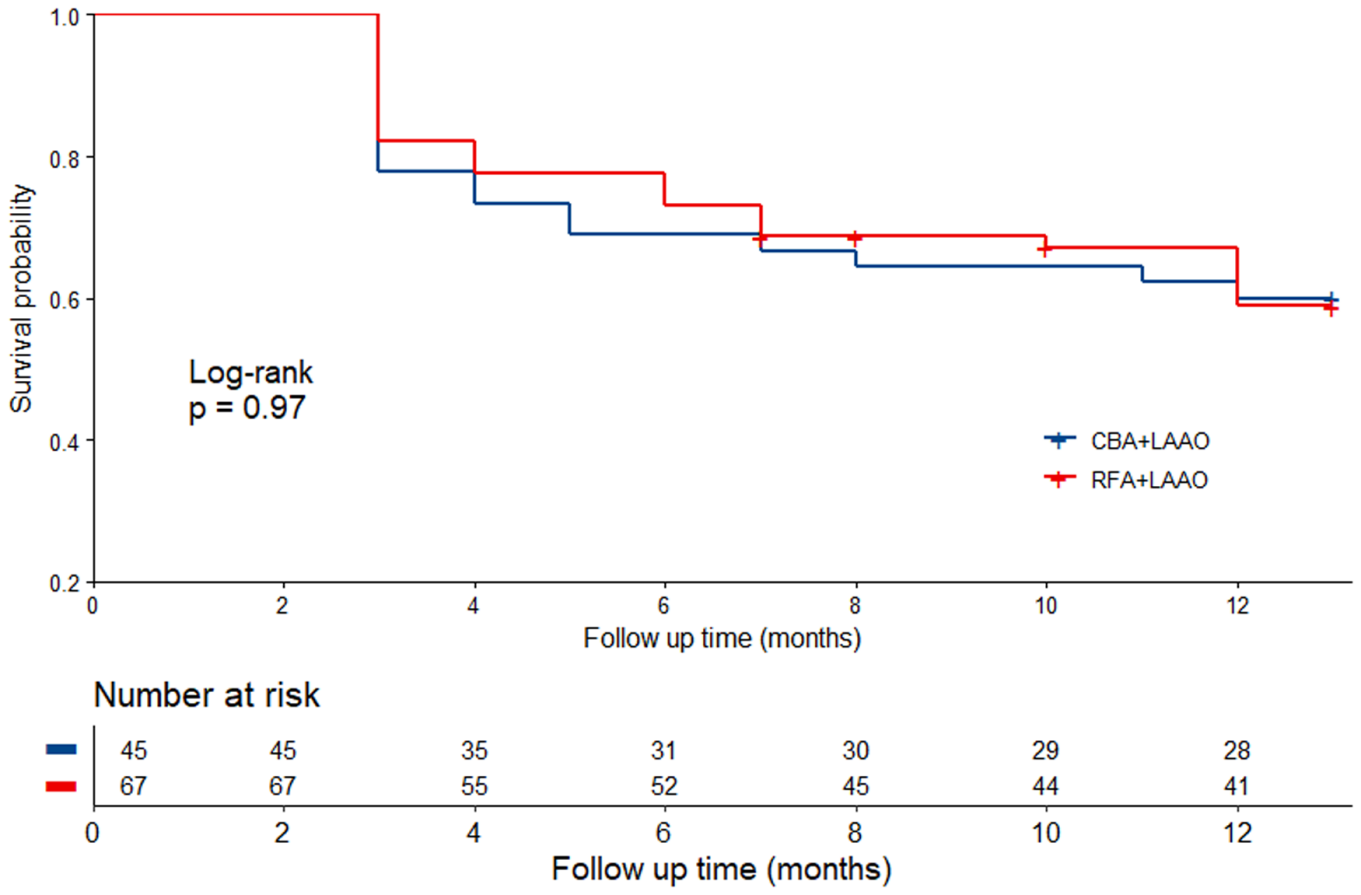
Time to arrhythmia recurrence in different groups. CBA, cryoballoon ablation; LAAO, left atrial appendage occlusion; RFA, radiofrequency ablation.

After an aggregate of 91.4 patient-years follow-up, 45 (40.2%) patients experienced arrhythmia recurrence, with 18 (40.0%) patients from group 1 and 27 (40.3%) patients from group 2 (log-rank *p* = 0.97). Time to arrhythmia recurrence in the whole cohort is shown in Figure 2.

### 3.6. End point analyses

After adjusting for a series of covariables, multivariable logistic regression showed that compared with LAAO combined with RFA, LAAO combined with CBA was not associated with more PDLs or safety outcomes (Table 5). Propensity score matching analysis showed the same results as well (Supplementary Tables S3–S5).

**Table 5:** End point analyses.

	Group 1	Group 2	OR or RR	95% CI	*P* value
**Peri-device leak**					
Univariable	15/45 (33.3)	25/67 (37.3)	0.840	0.380–1.857	0.667
Multivariable model 1			0.935	0.413–2.115	0.935
Multivariable model 2			0.814	0.313–2.116	0.672
Multivariable model 3*			1.162	0.492–2.746	0.733
**Safety outcomes**					
Univariable	3/45 (6.7)	5/67 (7.5)	0.886	0.201–3.906	0.873
Multivariable model 1			0.953	0.208–4.359	0.950
Multivariable model 2			0.633	0.086–4.648	0.653
Multivariable model 3*			0.861	0.177–4.185	0.853

OR, odd ratio; RR, relative risk; CI, confidence interval.

* Multivariable model 3 was adjusted for HASBLED score and left atrial diameter.

**Figure 3:**
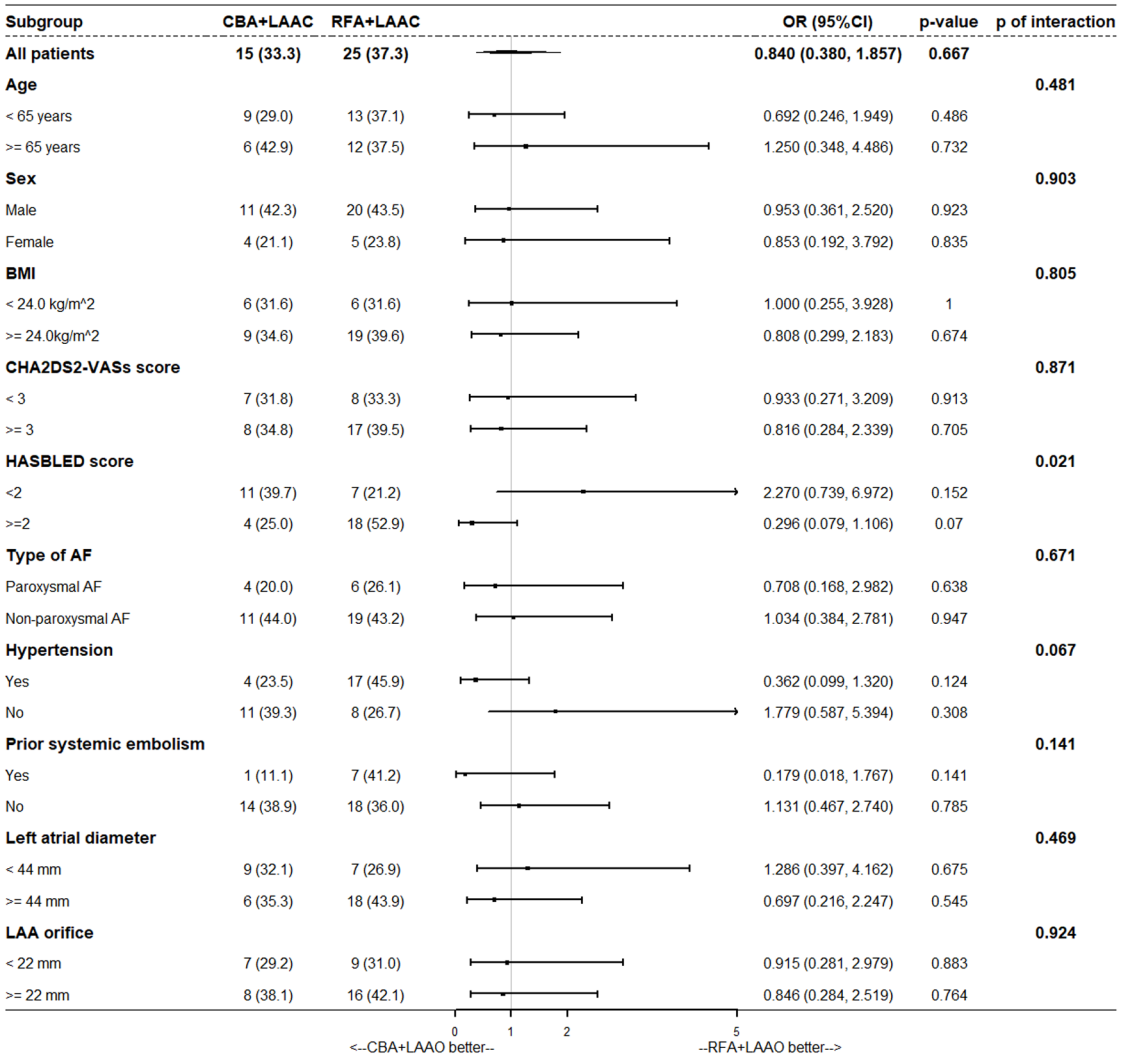
Subgroup analysis. BMI, body mass index; CBA, cryoballoon ablation; LAAO, left atrial appendage occlusion; RFA, radiofrequency ablation.

Subgroup analysis is shown in Figure 3. Results showed that the HASBLED score was a stratification variable (interaction *p* = 0.021); however, for each subgroup, the results were not significant (HASBLED score < 2, OR = 2.270, 95% CI: 0.739–6.972, *p* = 0.152; HASBLED score ≥ 2, OR = 0.296, 95% CI: 0.079–1.106, *p* = 0.070). Details in subgroup and threshold selection are shown in Supplementary Table S6 and Figure S1.

**Figure 4:**
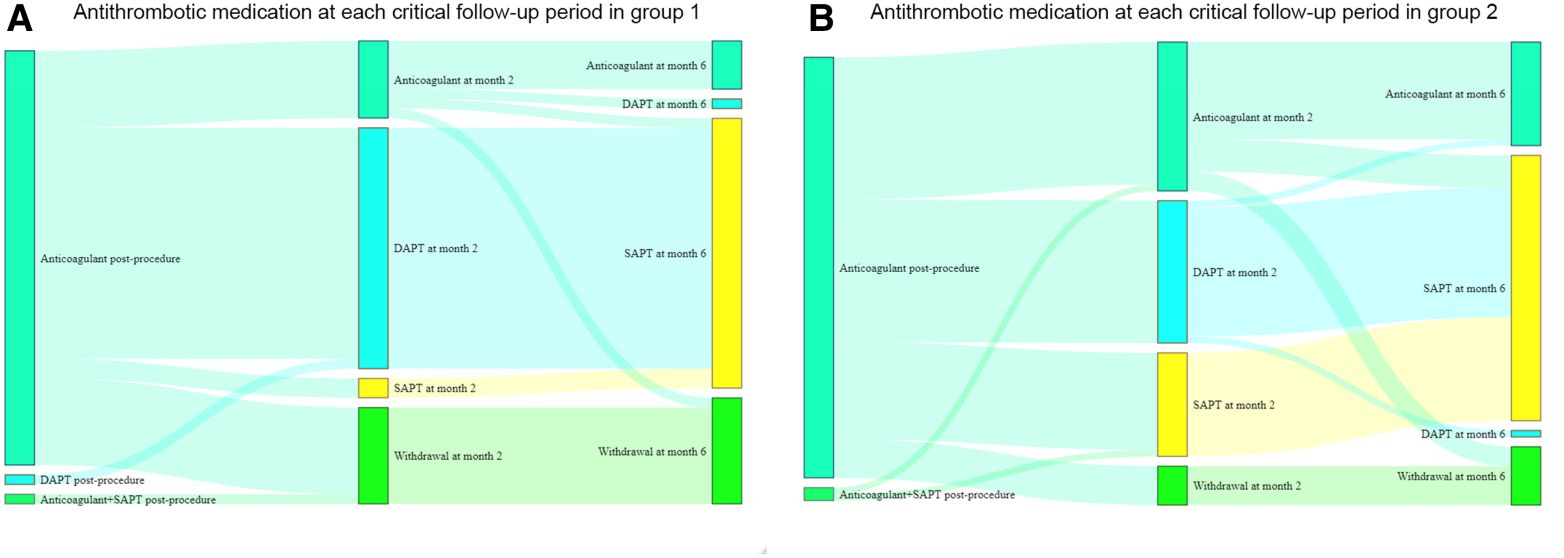
Antithrombotic medication at each critical follow-up points in different groups. DAPT, dual antiplatelet therapy; SAPT, single antiplatelet therapy.

Antithrombotic medication at each critical follow-up point is shown in Figure 4. In group 1, 10 (22.2%) patients discontinued antithrombotic medication from month 2, and 1 (2.2%) patients discontinued antithrombotic medication from month 6 (24.4% in total); 5 (11.1%) patients received long-term anticoagulant treatment. In group 2, 6 (9.0%) patients discontinued antithrombotic medication from month 2, and 3 (4.5%) patients discontinued antithrombotic medication from month 6 (13.4% in total); 15 (22.4%) patients received long-term anticoagulant treatment. Antithrombotic medication in patients with or without PDLs is shown in Supplementary Figure S2. Patients without PDLs were less likely to receive antithrombotic therapy.

## 4. Discussion

In the present study, we compared the efficacy and safety for CBA vs. RFA one-stop procedures. We found the following: (i) the CBA one-stop procedure yielded shorter procedure times; (ii) the incidence rates of PDLs, safety outcomes, and arrhythmia recurrence were comparable between the two methods; (iii) after adjusting for multiple covariables, similar risks for PDLs and safety outcomes were verified for both one-stop procedures; (iv) no significant differences between the two groups were shown by subgroup analysis; (v) follow-up safety outcomes were related to anticoagulant medication, and patients without PDLs were more likely to discontinue antithrombotic therapy. Together, these findings show that the CBA one-stop procedure is effective and safe and has unique advantages.

### 4.1. Peri-device leaks in combined procedures

Catheter ablation can cause coumadin ridge edema, which may affect the efficacy of LAAO. It is generally believed that for disc closure devices, coumadin ridge edema may cause larger PDL; for plug closure devices, coumadin ridge edema may have little effect on PDL due to its integrated design. Therefore, only patients who received a Watchman device were enrolled in this study to ensure consistency.

Multiple studies have shown that one-stop procedures do not increase the incidence of occlusion failure. Results for RFA combined with the LAAO one-stop procedure have been widely reported in previous studies. Phillips et al. reported a 5-year single-center study of the one-stop procedure where 100.0% of the patients successfully completed the LAAO procedure (13). In a long-term multicenter registry for the one-stop procedure, all 349 patients received successful device implantation, and only 7.4% had minor PDLs intra-procedure (15). A one-stop procedure subgroup study from the EWOLUTION and WASP registries showed that Watchman devices were successfully implanted in 99.3% of patients, and PDLs were not detected in 97.2% of patients; during follow-up, 98.2% of patients achieved successful occlusion, and only 2 patients had a PDL > 5 mm due to device displacement (14, 16). Chen et al. reported a single-center study of the one-stop procedure in which only 0.3% of patients experienced occlusion failure (1,114 patients in total) (17). There are few reports of PDL follow-up for the CBA one-stop procedure; Fassini et al. reported that all 45 patients receiving CBA combined with LAAO had successful implantation, and Ren et al. reported that only 1 of 72 patients experienced disc device occlusion failure (18, 19). Currently, there is no head-to-head comparison between CBA and RFA one-stop procedures. The results from our study reveal that the occlusion success rate is similar in both CBA and RFA one-stop procedures, as well as the follow-up PDL risk.

CBA and RFA have different coumadin ridge edema mechanisms. RFA causes tissue damage and necrosis through impedance or conduction heating and may be accompanied by bleeding; CBA directly destroys the myocardial structure and causes necrosis through rewarming (27, 28). A magnetic resonance imaging evaluation of myocardial damage after catheter ablation showed that CBA resulted in a similar magnitude of damage as RFA, but the resolution of CBA-related damage was more gradual (20). Our study shows that the incidence and risk of follow-up PDL were similar for both CBA and RFA one-stop procedures, suggesting that edema caused by these two mechanisms may have similar effects on occlusion procedures. Phillips et al. indicated that although RFA caused significant LAA orifice edema, the anchor area of the closure device was far from the edema area (13). Although the incidence of all PDL was similar, the >3 mm newly-developed PDL and progressed PDL were all significantly lower in group 1 than in group 2. However, the left atrial diameter (a risk factor of PDL) was also significantly smaller in group 1 than in group 2. Because of the low incidence of above two indexes in group 1, it has the limitation to performed multivariable logistic regression analysis. Larger scale studies are needed to evaluate whether ridge edema caused by CBA or RFA affects LAAO efficacy.

### 4.2. Safety outcomes of combined procedures

Previous studies have noted that compared with LAAO alone, one-stop procedures (including CBA and RFA one-stop procedures) do not increase the risk of peri-procedural SAEs (8). Multiple studies have shown that one-stop procedures can significantly reduce the risk of thromboembolism in high-risk AF patients. In a 35-month follow-up of 349 patients who underwent one-stop procedure, Wintgens et al. found that the annual incidence of ischemic stroke was only 0.7%, with a 78% reduction in absolute risk (15). A one-stop procedure subgroup from the EWOLUTION and WASP registries showed that the annual incidence of cerebral infarction was only 0.36%, and the absolute risk decreased by 93% (16). Fassini et al. showed that no SAEs occurred during the 24-month follow-up in 35 patients who received a one-stop procedure, and only one case of stroke was found in a longer follow-up involving more patients (18). Our results showed that the incidence rates of peri-procedural and follow-up adverse events for the one-stop procedures were both low and comparable to those reported in previous studies. These rates were also comparable between the two groups. During the 1-year follow-up, no death or ischemic stroke events were observed, and a device-related thrombus was detected in only 1 patient. Only 1 patient experienced major bleeding, which was related to an oral disease. These adverse events were associated with the use of anticoagulants, rather than procedures. The optimal antithrombotic treatment strategy after one-stop procedure remains unclear. In real world clinical practice, most LAAO patients may require additional catheter ablation for better rhythm control. Although the purposes of rhythm control and thrombosis prevention in the treatment of AF are not the same, there may be some overlap between them. Previous studies have shown that rhythm control can replace thromboprophylaxis to some extent, without increasing the risk of thromboembolism, and can significantly reduce the risk of bleeding in patients (29). In our study, regardless of whether they received the RFA or CBA one-stop procedure, patients without PDLs chose to discontinue antithrombotic drugs more frequently, as has been reported in previous studies. Wintgens et al. reported that by the end of follow-up, 6.5% of patients had discontinued antithrombotic drug use (15). Fassini et al. reported that 8 (16.3%) patients had discontinued using antithrombotic drugs 3 months post-procedure (18). Discontinuing anticoagulants can effectively prevent non-procedural hemorrhages, but early anticoagulant discontinuance may have certain risks. In this study, patients with device-related thrombi were associated with premature anticoagulant withdrawal. It has been suggested that platelets do not participate in endothelialization during LAAO; this process may be more closely related to antithrombin III (30). Therefore, it seems reasonable to discontinue antithrombotic drugs in those who have completed endothelialization. Previous studies have shown that PDLs may increase the risk of non-disabling stroke (21). In the present study, antithrombotic therapy was discontinued more frequently in patients without PDLs, regardless of whether they received CBA or RFA. The results fully support the safety of this strategy. A randomized controlled trial of standard regiments after Watchman occlusion vs. discontinuation of antithrombotic therapy at 6 months post-procedure is being conducted and is expected to provide strong evidence for clinical practice (31).

### 4.3. Catheter ablation in left atrial appendage occlusion populations

One of the advantages of CBA over RFA is that the procedure time is significantly shorter. High-power ablation is a relatively efficient RFA strategy in current clinical practice, but a randomized controlled trial revealed that the ablation time and the total procedure time were still significantly shorter for CBA than high-power ablation. In addition, the recurrence rates and risk of AF post-ablation were similar between the two ablation methods. These results suggest that CBA is an efficient ablation method (32). In our study, the CBA one-stop procedure was significantly superior to the RFA one-stop procedure in terms of total procedure time and ablation time, indicating that the CBA one-stop procedure has unique advantages in its indication population.

## 5. Limitations

The sample size of this study was relatively small, and the follow-up duration was short, which may not be conducive to observing the full range of clinical adverse events. In this study, both transesophageal echocardiography and cardiac computed tomography angiography were used for LAAO evaluation, and these data may not be directly comparable with previous studies. However, the incidence of follow-up PDL in the present study was in between the results of transesophageal echocardiography alone and cardiac computed tomography angiography alone (16, 33). Finally, the present study does provide direct head-to-head comparison results for the CBA one-stop procedure vs. LAAO alone.

## 6. Conclusion

Left atrial appendage occlusion combined with cryoballoon ablation has the same peri-device leak risk and safety outcomes as left atrial appendage occlusion combined with radiofrequency. The incidence of arrhythmia recurrence between procedures was similar as well, but the procedure time is significantly reduced. The combination of left atrial appendage occlusion and cryoballoon ablation with appropriate antithrombotic medication is efficacy and safety. Large-scale and long-term follow-up studies are warranted to verify our findings.

## Data Availability

The original contributions presented in the study are included in the article/**Supplementary Material**, further inquiries can be directed to the corresponding author.

## References

[1] Vaduganathan M Mensah GA Turco JV Fuster V Roth GA. The global burden of cardiovascular diseases and risk: a compass for future health. J Am Coll Cardiol. (2022) 80:2361–71. 10.1016/j.jacc.2022.11.005 36368511

[2] Hindricks G Potpara T Dagres N Arbelo E Bax JJ Blomström-Lundqvist C 2020 ESC guidelines for the diagnosis and management of atrial fibrillation developed in collaboration with the European association for cardio-thoracic surgery (EACTS). Eur Heart J. (2021) 42:373–498. 10.1093/eurheartj/ehaa612 32860505

[3] Steffel J Verhamme P Potpara TS Albaladejo P Antz M Desteghe L The 2018 European heart rhythm association practical guide on the use of non-vitamin K antagonist oral anticoagulants in patients with atrial fibrillation. Eur Heart J. (2018) 39:1330–93. 10.1093/eurheartj/ehy136 29562325

[4] Aberg H. Atrial fibrillation. I. A study of atrial thrombosis and systemic embolism in a necropsy material. Acta Med Scand. (1969) 185:373–9. 10.1111/j.0954-6820.1969.tb07351.x 5808636

[5] Reddy VY Sievert H Halperin J Doshi SK Buchbinder M Neuzil P Percutaneous left atrial appendage closure vs warfarin for atrial fibrillation: a randomized clinical trial. JAMA. (2014) 312:1988–98. 10.1001/jama.2014.15192 25399274

[6] Reddy VY Doshi SK Kar S Gibson DN Price MJ Huber K 5-Year Outcomes after left atrial appendage closure: from the PREVAIL and PROTECT AF trials. J Am Coll Cardiol. (2017) 70:2964–75. 10.1016/j.jacc.2017.10.021 29103847

[7] Osmancik P Herman D Neuzil P Hala P Taborsky M Kala P 4-Year Outcomes after left atrial appendage closure versus nonwarfarin oral anticoagulation for atrial fibrillation. J Am Coll Cardiol. (2022) 79:1–14. 10.1016/j.jacc.2021.10.023 34748929

[8] Su F Gao C Liu J Ning Z He B Liu Y Periprocedural outcomes associated with use of a left atrial appendage occlusion device in China. JAMA Netw Open. (2022) 5:e2214594. 10.1001/jamanetworkopen.2022.14594 35639378 PMC9157261

[9] Chen M Wang ZQ Wang QS Sun J Zhang PP Feng XF One-stop strategy for treatment of atrial fibrillation: feasibility and safety of combining catheter ablation and left atrial appendage closure in a single procedure. Chin Med J (Engl). (2020) 133:1422–8. 10.1097/CM9.0000000000000855 32433041 PMC7339144

[10] Kuck KH Brugada J Fürnkranz A Metzner A Ouyang F Chun KR Cryoballoon or radiofrequency ablation for paroxysmal atrial fibrillation. N Engl J Med. (2016) 374:2235–45. 10.1056/NEJMoa1602014 27042964

[11] Andrade JG Champagne J Dubuc M Deyell MW Verma A Macle L Cryoballoon or radiofrequency ablation for atrial fibrillation assessed by continuous monitoring: a randomized clinical trial. Circulation. (2019) 140:1779–88. 10.1161/CIRCULATIONAHA.119.042622 31630538

[12] Shi LB Rossvoll O Tande P Schuster P Solheim E Chen J. Cryoballoon vs. Radiofrequency catheter ablation: insights from Norwegian randomized study of PERSistent atrial fibrillation (NO-PERSAF study). Europace. (2022) 24:226–33. 10.1093/europace/euab281 35134151 PMC8824490

[13] Phillips KP Walker DT Humphries JA. Combined catheter ablation for atrial fibrillation and watchman® left atrial appendage occlusion procedures: five-year experience. J Arrhythm. (2016) 32:119–26. 10.1016/j.joa.2015.11.001 27092193 PMC4823577

[14] Phillips KP Pokushalov E Romanov A Artemenko S Folkeringa RJ Szili-Torok T Combining watchman left atrial appendage closure and catheter ablation for atrial fibrillation: multicentre registry results of feasibility and safety during implant and 30 days follow-up. Europace. (2018) 20:949–55. 10.1093/europace/eux183 29106523 PMC5982721

[15] Wintgens L Romanov A Phillips K Ballesteros G Swaans M Folkeringa R Combined atrial fibrillation ablation and left atrial appendage closure: long-term follow-up from a large multicentre registry. Europace. (2018) 20:1783–9. 10.1093/europace/euy025 29547906

[16] Phillips KP Romanov A Artemenko S Folkeringa RJ Szili-Torok T Senatore G Combining left atrial appendage closure and catheter ablation for atrial fibrillation: 2-year outcomes from a multinational registry. Europace. (2020) 22:225–31. 10.1093/europace/euz286 31665276

[17] Chen M Sun J Wang QS Zhang PP Li W Zhang R Long-term outcome of combined catheter ablation and left atrial appendage closure in atrial fibrillation patients. Int J Cardiol. (2022) 368:41–8. 10.1016/j.ijcard.2022.08.007 35952939

[18] Fassini G Gasperetti A Italiano G Riva S Moltrasio M Dello Russo A Cryoballoon pulmonary vein ablation and left atrial appendage closure combined procedure: a long-term follow-up analysis. Heart Rhythm. (2019) 16:1320–6. 10.1016/j.hrthm.2019.03.022 30928784

[19] Ren Z Zhang J Wang S Jia P Li X Zhang J Two-Year outcome from combining cryoballoon ablation and left atrial appendage closure: cLACBAC study. Front Cardiovasc Med. (2021) 7:610537. 10.3389/fcvm.2020.610537 33505994 PMC7829213

[20] Yamashita K Kholmovski E Ghafoori E Kamali R Kwan E Lichter J Characterization of edema after cryo and radiofrequency ablations based on serial magnetic resonance imaging. J Cardiovasc Electrophysiol. (2019) 30:255–62. 10.1111/jce.13785 30375090 PMC6363882

[21] Dukkipati SR Holmes DR Jr Doshi SK Kar S Singh SM Gibson D Impact of peridevice leak on 5-year outcomes after left atrial appendage closure. J Am Coll Cardiol. (2022) 80:469–83. 10.1016/j.jacc.2022.04.062 35902169

[22] Su WW Reddy VY Bhasin K Champagne J Sangrigoli RM Braegelmann KM Cryoballoon ablation of pulmonary veins for persistent atrial fibrillation: results from the multicenter STOP persistent AF trial. Heart Rhythm. (2020) 17:1841–7. 10.1016/j.hrthm.2020.06.020 32590151

[23] Yang B Jiang C Lin Y Yang G Chu H Cai H STABLE-SR (Electrophysiological substrate ablation in the left atrium during Sinus rhythm) for the treatment of nonparoxysmal atrial fibrillation: a prospective, multicenter randomized clinical trial. Circ Arrhythm Electrophysiol. (2017) 10:e005405. 10.1161/CIRCEP.117.005405 29141843

[24] Meier B Blaauw Y Khattab AA Lewalter T Sievert H Tondo C EHRA/EAPCI expert consensus statement on catheter-based left atrial appendage occlusion. EuroIntervention. (2015) 10:1109–25. 10.4244/EIJY14M08_18 25169595

[25] Enomoto Y Gadiyaram VK Gianni C Horton RP Trivedi C Mohanty S Use of non-warfarin oral anticoagulants instead of warfarin during left atrial appendage closure with the watchman device. Heart Rhythm. (2017) 14:19–24. 10.1016/j.hrthm.2016.10.020 27771552

[26] Mehran R Rao SV Bhatt DL Gibson CM Caixeta A Eikelboom J Standardized bleeding definitions for cardiovascular clinical trials: a consensus report from the bleeding academic research consortium. Circulation. (2011) 123:2736–47. 10.1161/CIRCULATIONAHA 21670242

[27] Harrison JL Jensen HK Peel SA Chiribiri A Grøndal AK Bloch LØ Cardiac magnetic resonance and electroanatomical mapping of acute and chronic atrial ablation injury: a histological validation study. Eur Heart J. (2014) 35:1486–95. 10.1093/eurheartj/eht560 24419806 PMC4048535

[28] Avitall B Kalinski A. Cryotherapy of cardiac arrhythmia: from basic science to the bedside. Heart Rhythm. (2015) 12:2195–203. 10.1016/j.hrthm.2015.05.034 26031374

[29] Yang WY Du X Jiang C He L Fawzy AM Wang L The safety of discontinuation of oral anticoagulation therapy after apparently successful atrial fibrillation ablation: a report from the Chinese atrial fibrillation registry study. Europace. (2020) 22:90–9. 10.1093/europace/euz235 31909431

[30] Rodés-Cabau J O'Hara G Paradis JM Bernier M Rodriguez-Gabella T Regueiro A Changes in coagulation and platelet activation markers following transcatheter left atrial appendage closure. Am J Cardiol. (2017) 120:87–91. 10.1016/j.amjcard.2017.03.253 28495432

[31] Chen M Wang Q Sun J Zhang PP Li W Mo BF Double-blind, placebo-controlled randomised clinical trial to evaluate the effect of ASPIRIN discontinuation after left atrial appendage occlusion in atrial fibrillation: protocol of the ASPIRIN LAAO trial. BMJ Open. (2021) 11:e044695. 10.1136/bmjopen-2020-044695 33722871 PMC7970210

[32] Pak HN Park JW Yang SY Kim TH Uhm JS Joung B Cryoballoon versus high-power, short-duration radiofrequency ablation for pulmonary vein isolation in patients with paroxysmal atrial fibrillation: a single-center, prospective, randomized study. Circ Arrhythm Electrophysiol. (2021) 14:e010040. 10.1161/CIRCEP.121.010040 34465132

[33] Galea R De Marco F Meneveau N Aminian A Anselme F Gräni C Amulet or watchman device for percutaneous left atrial appendage closure: primary results of the SWISS-APERO randomized clinical trial. Circulation. (2022) 145:724–38. 10.1161/CIRCULATIONAHA.121.057859 34747186

