# Supplementary material for: Left atrial appendage occlusion combined with cryoballoon or radiofrequency ablation: One-year follow-up comparison
Source: Front Cardiovasc Med. 2023 Apr 26;10:1153158. doi: 10.3389/fcvm.2023.1153158 (PMC10169712; doi:10.3389/fcvm.2023.1153158)
Supplement: Supplementary file 1 [file Datasheet1.docx]

**Supplementary tables**

**Supplementary Table 1. PASS principle**

**Supplementary Table 2. Ablation details**

**Supplementary Table 3. Patient characteristics at baseline after propensity score matching**

Data were described as mean ± SD, median (IQR) or n (%).

AF, atrial fibrillation; LDL-C, low density lipoprotein cholesterol; HDL-C, high density lipoprotein cholesterol; TG, total triglycerides.

**Supplementary Table 4. Periprocedural characteristics after propensity score matching**

Data were described as mean ± SD, median (IQR) or n (%).

**Supplementary Table 5. Clinical outcomes after propensity score matching**

OR, odd ratio; CI, confidence interval; PDL, peri-device leak.

**Supplementary Table 6. Potential risk factors for PDL**

PDL, peri-device leak; OR, odd ratio; CI, confidence interval. BMI, body mass index; LDL-C, low density lipoprotein cholesterol; HDL-C, high density lipoprotein cholesterol; TG, total triglycerides; LAA, left atrial appendage.

**Supplementary figure and figure legends**

**Supplementary Figure 1. Appropriate thresholds determination for PDL**

OR, odd ratio; CI, confidence interval; CBA, cryoballoon ablation; LAAO, left atrial appendage occlusion; RFA, radiofrequency ablation.

**Supplementary Figure 2. Antithrombotic medication in patients with or without PDLs**

PDL, peri-device leak; DAPT, dual antiplatelet therapy; SAPT, single antiplatelet therapy.

**Supplementary Table 1. PASS principle**

| Position (P) | The maximum diameter of the closure device is just at or slightly away from the left atrial appendage orifice plane (shoulder exposure ≤ 1/3). |
| --- | --- |
| Anchor (A) | The position of the closure device is stable, and the closure device moves in tandem with the left atrial appendage. |
| Size (S) | Compression ratio 8 – 30% |
| Seal (S) | Peri-device leak ≤ 5-mm |

**Supplementary Table 2. Ablation details**

|  | Group 1 (n = 45) | Group 2 (n = 67) |
| --- | --- | --- |
| Pulmonary vein isolation only, n (%) | 45 (100.0) | 27 (40.3) |
| Substrate modification, n (%) | 0 (0.0) | 40 (59.7) |
| Left atrial roof, n (%) | 0 (0.0) | 25 (37.3) |
| Left atrial posterior wall, n (%) | 0 (0.0) | 14 (20.9) |
| Left atrial anterior wall, n (%) | 0 (0.0) | 11 (16.4) |
| Mitral isthmus, n (%) | 0 (0.0) | 16 (23.9) |
| Coronary sinus, n (%) | 0 (0.0) | 9 (13.4) |
| Left atrial appendage, n (%) | 0 (0.0) | 1 (1.5) |
| Cavotricuspid isthmus, n (%) | 0 (0.0) | 13 (19.4) |
| Cristae terminalis, n (%) | 0 (0.0) | 1 (1.5) |
| Slow pathway, n (%) | 0 (0.0) | 1 (1.5) |
| Vein of Marshall ethanol infusion, n (%) | 0 (0.0) | 8 (11.9) |

**Supplementary Table 3. Patient characteristics at baseline after propensity score matching**

|  | Group 1 (n = 26) | Group 2 (n = 26) | P value |
| --- | --- | --- | --- |
| **Demographic characteristics** | | | |
| Age, years | 62.5 ± 8.1 | 62.6 ± 8.0 | 0.973 |
| Height, m | 166.9 ± 9.1 | 167.6 ± 7.4 | 0.765 |
| Weight, kg | 69.9 ± 12.3 | 69.4 ± 11.8 | 0.873 |
| Body mass index, kg/m^2^ | 25.0 ± 3.5 | 24.6 ± 3.3 | 0.692 |
| **AF overview** | | | |
| Paroxysmal, n (%) | 10 (38.5) | 13 (50.0) | 0.577 |
| Non-paroxysmal, n (%) | 16 (61.5) | 13 (50.0) | 0.577 |
| Time to first diagnosis, months | 18 (4, 72) | 12 (2, 36) | 0.503 |
| **Commodities or risk factors** | | | |
| CHA_2_DS_2_-VASc score | 3 (1, 4) | 3 (2, 3) | 0.910 |
| HASBLED score | 1 (0, 2) | 1 (0, 2) | 0.969 |
| Heart failure, n (%) | 5 (19.2) | 3 (11.5) | 0.703 |
| Hypertension, n (%) | 10 (38.5) | 11 (42.3) | 1.000 |
| Age ≥ 75 years, n (%) | 0 (0.0) | 3 (11.5) | 0.235 |
| Diabetes, n (%) | 2 (7.7) | 2 (7.7) | 1.000 |
| Systemic embolism, n (%) | 4 (15.4) | 5 (19.2) | 1.000 |
| Vascular disease, n (%) | 23 (84.6) | 19 (73.1) | 0.499 |
| Coronary artery disease, n (%) | 3 (11.5) | 4 (15.4) | 1.000 |
| Age 65 – 74 years, n (%) | 11 (42.3) | 7 (26.9) | 0.382 |
| Female, n (%) | 15 (57.7) | 18 (69.2) | 0.565 |
| **Examinations** | | | |
| Left atrial diameter, mm | 43.2 ± 4.7 | 42.9 ± 6.2 | 0.861 |
| Ejection fraction, % | 57.5 (56.0, 60.0) | 57.0 (56.0, 60.0) | 0.825 |
| Pathological regurgitation |  |  |  |
| Mitral regurgitation, n (%) | 3 (11.5) | 5 (19.2) | 0.703 |
| Tricuspid regurgitation, n (%) | 6 (23.1) | 7 (26.9) | 1.000 |
| HbO_2_, g/L | 144.8 ± 13.8 | 149.3 ± 15.0 | 0.269 |
| Alanine transaminase, U/L | 20.5 (16.0, 31.0) | 22.0 (16.0, 29.0) | 0.700 |
| Aspartate aminotransferase, U/L | 21.5 (19.0, 25.0) | 20.0 (16.0, 25.0) | 0.349 |
| NT-proBNP, pg/ml | 685.7 (348.7, 1080.0) | 488.5 (139.8, 959.6) | 0.241 |
| Serum Ca^+^, mmol/L | 2.2 ± 0.1 | 2.3 ± 0.1 | 0.357 |
| Glucose, mmol/L | 5.5 (4.9, 6.3) | 5.6 (5.1, 6.8) | 0.337 |
| LDL-C, mmol/L | 1.9 (1.6, 2.5) | 2.0 (1.4, 2.9) | 0.964 |
| HDL-C, mmol/L | 1.1 (1.0, 1.3) | 1.1 (0.9, 1.4) | 0.660 |
| TG, mmol/L | 1.1 (0.9, 1.6) | 1.2 (0.9, 2.2) | 0.464 |

Data were described as mean ± SD, median (IQR) or n (%).

AF, atrial fibrillation; LDL-C, low density lipoprotein cholesterol; HDL-C, high density lipoprotein cholesterol; TG, total triglycerides.

**Supplementary Table 4. Periprocedural characteristics after propensity score matching**

|  | Group 1 (n = 26) | Group 2 (n = 26) | P value |
| --- | --- | --- | --- |
| Left atrial appendage morphology |  |  | 0.825 |
| Cauliflower, n (%) | 19 (73.1) | 18 (69.2) | – |
| Chicken wing, n (%) | 3 (11.5) | 4 (15.4) | – |
| Reversed chicken wing, n (%) | 1 (3.8) | 0 (0.0) | – |
| Windsock, n (%) | 2 (7.7) | 1 (3.8) | – |
| Cactus, n (%) | 1 (3.8) | 3 (11.5) | – |
| Appendage ostia diameter, mm | 21.6 ± 3.3 | 21.5 ± 2.5 | 0.889 |
| Appendage depth, mm | 22.8 ± 4.7 | 22.3 ± 3.5 | 0.691 |
| Redo ablation, n (%) | 0 (0.0) | 2 (7.7) | 0.490 |
| Device size, mm | 27 (24, 30) | 27 (27, 27) | 0.724 |
| 21 mm | 6 (23.1) | 3 (11.5) | 0.465 |
| 24 mm | 3 (11.5) | 5 (19.2) | 0.703 |
| 27 mm | 7 (26.9) | 12 (46.2) | 0.249 |
| 30 mm | 9 (34.6) | 6 (23.1) | 0.541 |
| 33 mm | 1 (3.8) | 0 (0.0) | 1.000 |
| Device reselection, n (%) | 1 (3.8) | 1 (3.8) | 1.000 |
| Redeployment, n (%) | 8 (30.8) | 11 (42.3) | 0.565 |
| 1, n (%) | 6 (23.1) | 9 (34.6) | 0.541 |
| ≥2, n (%) | 2 (7.7) | 2 (7.7) | 1.000 |
| Peri-device leak, n (%) | 7 (26.9) | 3 (11.5) | 0.291 |
| Jet < 3-mm, n (%) | 6 (23.1) | 2 (7.7) | 0.248 |
| Jet 3 – 5-mm, n (%) | 1 (3.8) | 1 (3.8) | 1.000 |
| Total time spent, mins | 117.0 (100.0, 143.0) | 219.5 (197.0, 258.0) | 0.000 |
| Ablation time spent, mins | 140.0 (120.0, 165.0) | 240.0 (215.0, 280.0) | 0.000 |
| In hospital post-procedure, days | 2 (2, 3) | 2 (2, 3) | 0.901 |

Data were described as mean ± SD, median (IQR) or n (%).

**Supplementary Table 5. Clinical outcomes after propensity score matching**

|  | Group 1  (n = 26) | Group 2  (n = 26) | OR (95%CI) | P value |
| --- | --- | --- | --- | --- |
| **Primary efficacy endpoints** | | | | |
| PDL | 12 (46.2) | 7 (26.9) | 2.327 (0.729, 7.421) | 0.249 |
| Newly developed PDL | 5 (19.2) | 4 (15.4) | 1.310 (0.309, 5.551) | 1.000 |
| Persistent PDL | 7 (26.9) | 3 (11.5) | 2.825 (0.641, 12.442) | 0.291 |
| Progressed PDL | 6 (23.1) | 5 (19.2) | 1.260 (0.331, 4.790) | 1.000 |
| **Safety endpoints** | | | | |
| Total complications | 2 (7.7) | 3 (11.5) | 0.639 (0.098, 4.180) | 1.000 |
| Peri-procedural major bleeding | 0 (0.0) | 2 (7.7) | NA | 0.490 |
| Hematoma | 1 (3.8) | 1 (3.8) | 1.000 (0.059, 16.890) | 1.000 |
| Pericardial effusion | 1 (3.8) | 0 (0.0) | NA | 1.000 |
| Follow-up major bleeding | 0 (0.0) | 1 (3.8) | NA | 1.000 |

OR, odd ratio; CI, confidence interval; PDL, peri-device leak.

**Supplementary Table 6. Potential risk factors for PDL**

|  | B | OR | 95%CI | P value |
| --- | --- | --- | --- | --- |
| Age per 1 years | 0.005 | 1.005 | 0.963 – 1.049 | 0.816 |
| Female | -0.957 | 0.384 | 0.160 – 0.923 | 0.032 |
| BMI per 1 kg/m^2^ | 0.041 | 1.042 | 0.920 – 1.179 | 0.520 |
| Heart failure | 0.578 | 1.782 | 0.974 – 3.259 | 0.061 |
| Hypertension | -0.080 | 0.923 | 0.514 – 1.658 | 0.788 |
| Diabetes | 0.542 | 1.720 | 0.828 – 3.572 | 0.146 |
| Systemic embolism | 0.383 | 1.466 | 0.769 – 2.796 | 0.245 |
| Vascular disease | 0.247 | 1.280 | 0.596 – 2.749 | 0.527 |
| Coronary artery disease | 0.293 | 1.341 | 0.692 – 2.596 | 0.385 |
| Left atrial diameter per 1 mm | 0.077 | 1.080 | 1.022 – 1.140 | 0.006 |
| Ejection fraction per 1% | -0.004 | 0.996 | 0.944 – 1.051 | 0.897 |
| NT-proBNP per 1 pg/ml | 0.000 | 1.000 | 1.000 – 1.001 | 0.055 |
| Glucose per 1 mmol/L | 0.034 | 1.034 | 0.868 – 1.232 | 0.707 |
| LDL-C per 1 mmol/L | -0.277 | 0.758 | 0.518 – 1.110 | 0.154 |
| HDL-C per 1 mmol/L | -0.500 | 0.606 | 0.213 – 1.729 | 0.349 |
| TG per 1 mmol/L | -0.158 | 0.854 | 0.530 – 1.375 | 0.516 |
| LAA orifice per 1 mm | 0.054 | 1.055 | 0.941 – 1.183 | 0.357 |

PDL, peri-device leak; OR, odd ratio; CI, confidence interval. BMI, body mass index; LDL-C, low density lipoprotein cholesterol; HDL-C, high density lipoprotein cholesterol; TG, total triglycerides; LAA, left atrial appendage.


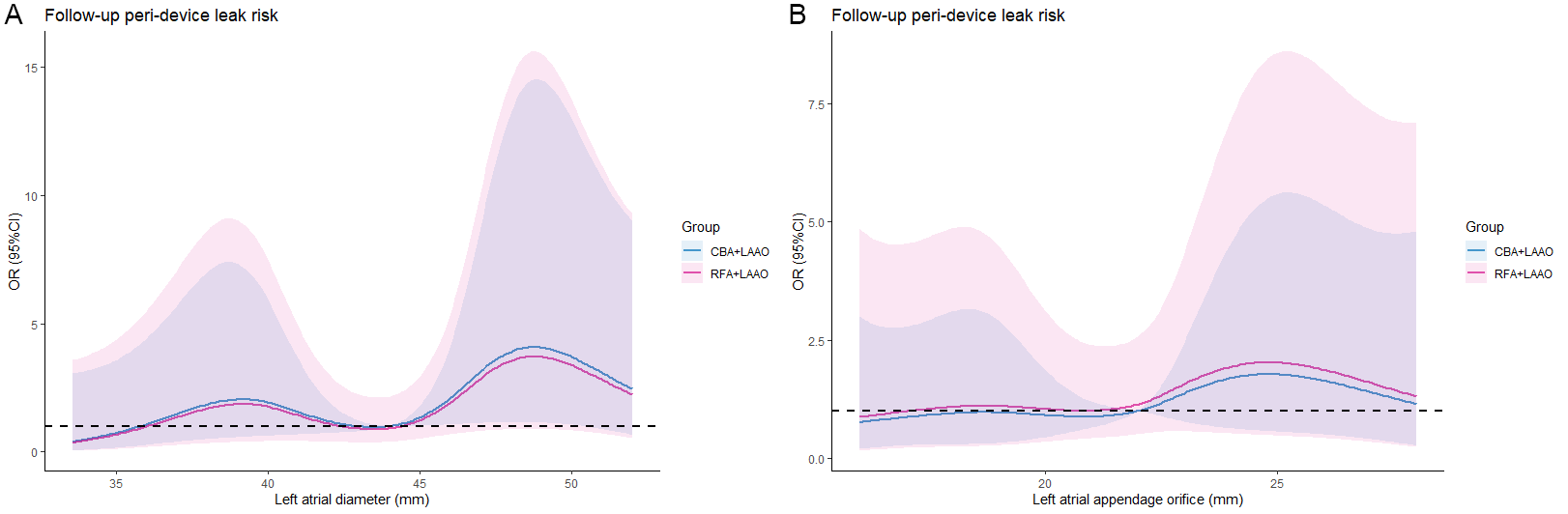


**Supplementary Figure 1. Appropriate thresholds determination for PDL**

OR, odd ratio; CI, confidence interval; CBA, cryoballoon ablation; LAAO, left atrial appendage occlusion; RFA, radiofrequency ablation.


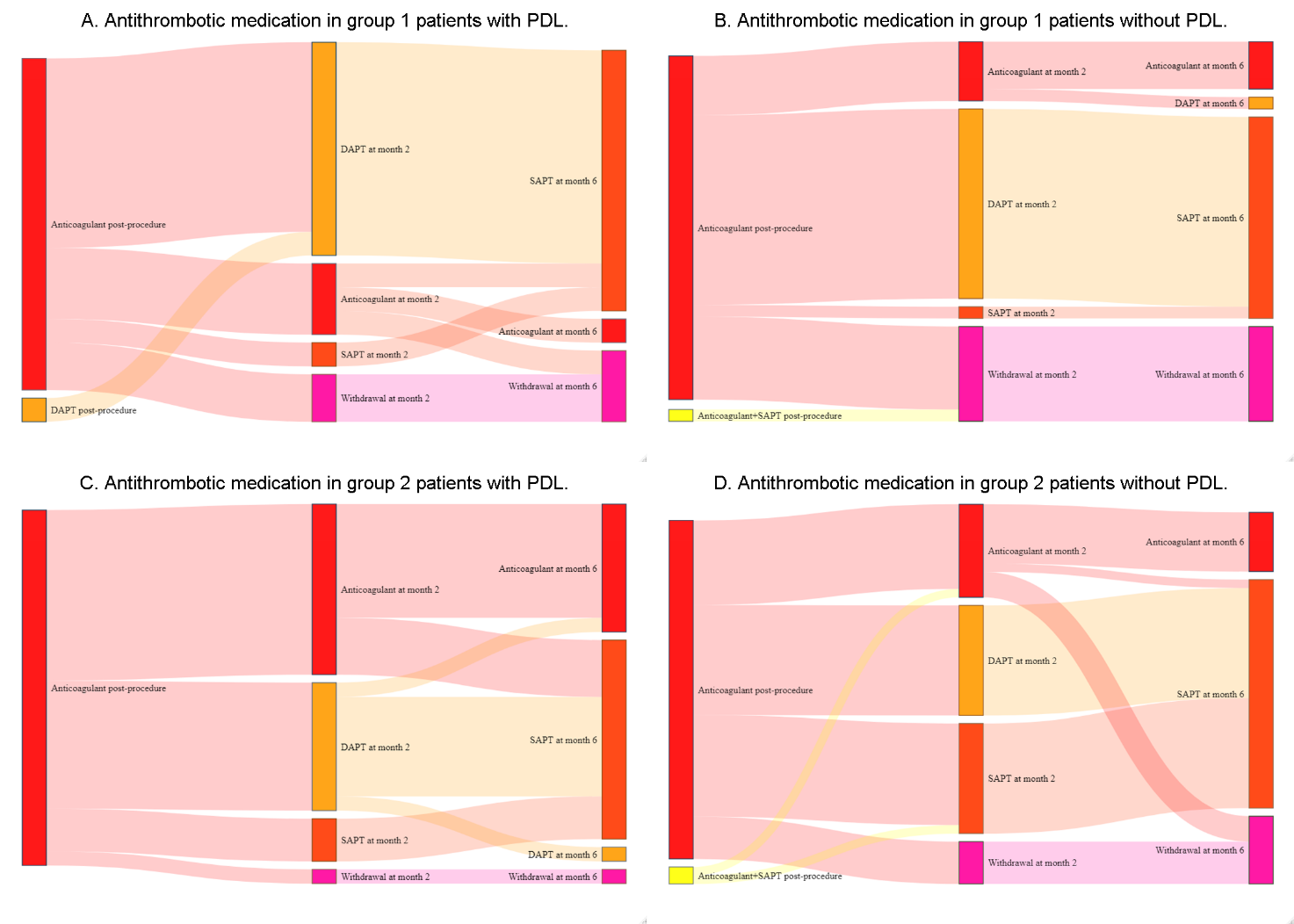


**Supplementary Figure 2. Antithrombotic medication in patients with or without PDLs**

PDL, peri-device leak; DAPT, dual antiplatelet therapy; SAPT, single antiplatelet therapy.
